# Phenotypic and functional abnormalities of bone marrow-derived dendritic cells in systemic lupus erythematosus

**DOI:** 10.1186/ar3018

**Published:** 2010-05-18

**Authors:** Ying J Nie, Mo Y Mok, Godfrey CF Chan, Albert W Chan, Ou Jin, Sushma Kavikondala, Albert KW Lie, Chak S Lau

**Affiliations:** 1 Department of Medicine, Li Ka Shing Faculty of Medicine, The University of Hong Kong, 21 Sassoon Road, Pokfulam, Hong Kong, PR China; 2 Department of Paediatrics & Adolescent Medicine, Li Ka Shing Faculty of Medicine, The University of Hong Kong, 21 Sassoon Road, Pokfulam, Hong Kong, PR China

## Abstract

**Introduction:**

Systemic lupus erythematosus (SLE) is an autoimmune disease characterized by autoreactive T and B cells, which are believed to be secondary to deficient dendritic cells (DCs). However, whether DC abnormalities occur during their development in the bone marrow (BM) or in the periphery is not known.

**Methods:**

Thirteen patients with SLE and 16 normal controls were recruited. We studied the morphology, phenotype, and functional abilities of bone marrow-derived dendritic cells (BMDCs) generated by using two culture methods: FMS-like tyrosine kinase 3 (Flt3)-ligand (FL) and granulocyte-macrophage colony-stimulating factor (GM-CSF) plus interleukin-4 (IL-4), respectively.

**Results:**

BMDCs induced by FL exhibited both myeloid (mDC) and plasmacytoid DC (pDC) features, whereas GM-CSF/IL-4 induced mDC generation. Substantial phenotypic and functional defects of BMDCs were found from patients with SLE at different stages of cell maturation. When compared with healthy controls, SLE immature BM FLDCs expressed higher levels of CCR7. Both immature and mature SLE BM FLDCs expressed higher levels of CD40 and CD86 and induced stronger T-cell proliferation. SLE BM mDCs expressed higher levels of CD40 and CD86 but lower levels of HLA-DR and a lower ability to stimulate T-cell proliferation when compared with control BM mDCs.

**Conclusions:**

Our data are in accordance with previous reports that suggest that DCs have a potential pathogenic role in SLE. Defects of these cells are evident during their development in BM. BM mDCs are deficient, whereas BM pDCs, which are part of BM FLDCs, are the likely culprit in inducing autoimmunity in SLE.

## Introduction

Systemic lupus erythematosus (SLE) is a multisystemic autoimmune disease characterized by autoreactive T and B cells [1,2]. Dendritic cells (DCs), the most effective antigen-presenting cells (APCs), are capable of activating naïve T cells and initiating T-cell responses. DCs have been hypothesized to play an important role in the pathogenesis of SLE [3,4].

DCs are developed in the bone marrow (BM), released into the circulation, and subsequently home to many tissues. The function of DCs varies according to their stage of maturity. Immature DCs are capable of capturing and processing antigens (Ags). After migration to the lymphoid organs, where they become mature, their ability to capture and process Ags decreases, whereas that for Ag presentation increases [5]. After maturation, DCs are capable of inducing the differentiation of naïve T cells into T-helper cells [6] with increased expression of adhesion molecules and cytokine receptors and cytokine production [7,8]. Activation of T cells requires two signals, the engagement of the T-cell receptor/CD3 complex with the antigenic peptide presented by the major histocompatibility complex (MHC), and the presence of co-stimulatory molecules and their ligands [6]. DCs could supply both signals for T-cell activation.

Two subsets of peripheral DCs have been identified in humans on the basis of their expression of CD11c: CD11c^+ ^myeloid DCs (mDCs) and CD11c^- ^plasmacytoid DCs (pDCs) [6,9,10]. Priming naïve T cells through Ag capture and presentation is the unique property of mDCs, whereas pDCs are inefficient in capturing Ag at all stages of development [11]. The site of distribution of the two subsets of DCs is different, too. mDCs are located mainly in the skin and mucosal tissues. Conversely, pDCs exist mainly within lymphoid tissues and may therefore be the major subset of APCs that recognize self-Ag and are responsible for immune tolerance [12].

In SLE, abnormalities in peripheral blood-isolated DCs, monocyte-derived DCs, and mouse BM-derived DCs have been reported [3,7,8,13,14]. All of these studies have indicated a crucial role of DCs in the pathogenesis of SLE through either a deficiency in sustaining peripheral tolerance to self-Ag or an increased susceptibility to infection. SLE serum has also been shown to induce DC generation, suggesting that some of the observed DC functional abnormalities may be acquired [15]. Whether SLE DC abnormalities occur during their development within the BM or as a result of microenvironmental changes or Ag capture in the peripheral blood and tissues, or both, remains unknown.

Two methods have been used to generate BM DCs (BMDCs). One uses culture of the BM cells in FMS tyrosine kinase 3 (Flt3)-ligand (FL), whereas the other uses granulocyte-macrophage colony-stimulating factor (GM-CSF) plus interleukin-4 (IL-4) to induce DC generation. Treatment of mouse BM with FL results in the expansion of both mDCs and pDCs, whereas GM-CSF/IL-4 treatment favors only the production of mDCs. Thus far, no culture methods have been identified that will generate pDCs alone from BM *in vitro*. The primary aim of this study was to explore whether FL- or GM-CSF/IL-4-generated BMDCs from patients with SLE were abnormal when compared with healthy controls. We analyzed the morphology, phenotype and functional ability of these DCs at different stages of development.

## Materials and methods

### Patients and controls

Patients who fulfilled the American College of Rheumatology classification criteria for SLE [16] were recruited from the Rheumatology Clinic of Queen Mary Hospital, Hong Kong. They had either cytopenia or fever requiring BM examination as part of their clinical investigations. The Systemic Lupus Erythematosus Disease Activity Index (SLEDAI) was used as a measure of overall disease activity [17]. Active disease was defined by an SLEDAI score of ≥ 6. None of the patients in this report had fever secondary to an underlying infection. Control subjects were BM donors of the Queen Mary Hospital Bone Marrow Transplantation Program. This study was approved by the Hong Kong University/Hong Kong West Cluster Institutional Review Board. A written informed consent was obtained from all subjects.

### Generation of BM-derived immature and mature DCs

DCs were obtained according to the methods reported previously, with some modifications [18]. In brief, human iliac crest BM cells (BMCs) were freshly aspirated from SLE patients or from BM donors. They were then isolated by Ficoll-Hypaque gradients. The BMCs used for DC culture were depleted of CD3^+ ^cells by anti-CD3 mAb-coated magnetic beads (Miltenyi Biotech Inc., Sunnyvale, CA, USA). The medium for DC generation consisted of RPMI-1640 supplemented with 10% fetal bovine serum (FBS), 100 U/ml penicillin, and 100 μg/ml streptomycin (Sigma Chemical, San Diego, CA). Aliquots of 2 × 10^6 ^cells were placed into six-well plates in culture medium containing 80 ng/ml FL (PharMingen, San Diego, CA, USA) or 20 ng/ml of GM-CSF (Biosource, Camarillo, CA, USA) plus 20 ng/ml IL-4 (PharMingen, San Diego, CA, USA). On day 4 or 5, culture medium was replaced with fresh medium.

After 8 days, nonadherent cells were harvested and washed once, and 1 × 10^6 ^cell aliquots were then transferred into the wells of additional six-well plates and were cultured with fresh medium for 3 additional days. Cells harvested from this culture were designated immature DC-enriched population. We found that FL cultured BMDCs exhibited features of both mDCs and pDCs (designated BM FLDCs), whereas GM-CSF/IL-4-cultured BMDCs exhibited features of mDCs (BM mDCs). To promote BMDC maturation, immature BM FLDCs were cultured for an additional 2 days with 80 ng/ml FL, 2 μmol/L oligodeoxynucleotide [ODN] containing unmethylated CpG motifs(CpG ODN)2006 and 2 μmol/L CpG ODN 2216 (InvivoGen, San Diego, CA, USA), 50 ng/ml tumor necrosis factor (TNF)-α (PharMingen), and 25 ng/ml lipopolysaccharide (LPS). Immature BM mDCs were cultured for an additional 3 days with 50 ng/ml TNF-α, 25 ng/ml LPS, 20 ng/ml GM-CSF, and 20 ng/ml IL-4 to become mature BM mDCs.

### Determination of cell morphology

Of the cells, 1 × 10^5 ^were centrifuged onto microscope slides with Cytopro 7620 (Wescor Inc., Provo, Utah, USA), stained with May-Grunwald-Giemsa solution and analyzed with light microscopy (Olympus, Tokyo, Japan).

### Phenotypic analysis of BM-derived immature and mature FLDCs and mDCs

Cells were incubated with 20 μl of either anti-CD3-FITC, anti-CD19-FITC, anti-CD34-FITC, anti-CD40-FITC, anti-HLA-DR-FITC, anti-DC-SIGN-FITC, anti-CD83-PE, anti-CD86-PE, anti-CD45RA-PE, anti-CD123-PE-CY5, anti-CD80-PE-CY5, or anti-CD11c-PE-CY5 (PharMingen) for 30 minutes. After washing to remove excess antibodies, the cells were analyzed with FACScan Immunocytometry (BD Pharmingen). Appropriate isotype-matched control antibodies were included as negative controls.

### IFN-α production assays

Supernatants of immature and mature BM FLDC and mDC cultures were examined for the production of interferon (IFN)-α by using the human IFN-α ELISA kit (Invitrogen Corporation, San Diego, CA, USA) according to the manufacturer's instructions. Five normal donors and three patients with SLE were studied.

### Proliferation assays

Allogeneic T cells were negatively isolated from normal donors' peripheral blood mononuclear cells (PBMCs) by using a Pan T-cell isolation Kit (Miltenyi Biotech, Gladbach, Germany), which yielded a purity of >95%, as assessed by CD3 expression. These purified T cells were then used as responder cells (Rs) in all subsequent proliferation assays. Before T-cell co-cultures, BMDCs were treated with mitomycin C. Allogeneic mitomycin C-treated BMDC-enriched populations were used as stimulators (Ss). Mitomycin C is an antitumoral antibiotic that has the ability to inhibit proliferation without affecting the viability of the feeder cells in long-term culture assays, thus reducing the interference of continued growth of these cells on the proliferation of the co-cultured responder cells [19]. Cell cultures were prepared with 1 × 10^5 ^T cells/well and 5 × 10^4 ^BMDCs/well (the R/S ratio is 2:1) in a 96-well plate, incubated for 4 days in 5% CO_2 _at 37°C, pulsed with 0.5 μCi ^3^H-thymidine (^3^H-TdR) for 16 hours, and then harvested and counted for radioactivity by using a beta scintillation counter (Packard Instruments, Chicago, IL, USA). Results are expressed as median counts per minute (cpm) of triplicate samples.

### Statistical methods

Statistical analysis was performed by using the unpaired two-tailed Student's *t *test with Microsoft Excel computer software program (Microsoft Corporation, Redmond, WA, USA).

## Results

### Subjects

Thirteen patients with SLE, all women, aged 26~57 (mean, 43 ± 9.5) years, were studied. Nine of 13 patients had active disease (SLEDAI ≥ 6). A summary of the clinical details of these patients is shown in Table 1. Sixteen healthy subjects, six male and 10 female, were recruited as controls. They were aged from 23~60 (mean, 45 ± 11) years.

**Table 1:** Clinical and laboratory characteristics of the SLE patients studied

Case	Current treatment	WBC (×10^9^/L)	Hb (g/dL)	Plt (×10^9^/L)	Lym (×10^9^/L)	Anti-dsDNA	Serum C3	Serum C4	24-hour UP	SLE-DAI
1	HCQ 200 mg/d	3.8	16.3	23	1.3	<5	54	13	0.24	3
2	Pred 5 mg/d, HCQ 200 mg/d	2.04	8.6	352	1.04	18	80	15	NA	1
3	Pred 12.5 mg/d, MMF 3 mg/d	10.55	9.2	281	0.45	34	72	24	6.57	6
4	HCQ 300 mg/d	2.38	12.1	149	0.64	127	29	7.1	<0.06	16
5	Pre 15 mg/d, HCQ 200 mg/d, Aza 100 mg/d	0.8	8.9	67	.3	19	94	19	0.18	6
6	Pred 20 mg/d, HCQ 400 mg/d	1.93	11.5	131	0.49	54	36	2.9	NA	15
7	Pred 7.5 mg/d, Aza 50 mg/d, MMF 1 mg/d	2.3	9.0	70	0.2	150	27	9.9	0.26	15
8	Pred 20 mg/d	4.9	10.4	207	1.4	9	67	13	NA	6
9	HCQ 200 mg/d	2.06	7.4	94	0.82	85	31	5.3	3.63	13
10	Pred 12.5 mg/d MMF 3 g/d	6.0	12.0	74	0.6	45	58	27	0.11	15
11	Pred 50 mg/d	3.54	9.4	47	0.9	29	101	34	NA	2
12	Pred 15 mg/d, HCQ 200 mg/d, Aza 100 mg/d	5.21	11.6	31	0.25	12	109	23	NA	1
13	HCQ 300 mg/d	4.1	9.5	92	1.2	>450	26	3.7	2.4	14

F, female; Pred, prednisolone;HCQ, hydroxychloroquine;Aza, azathioprine;MMF, mycophenolate mofetil;Hb, hemoglobin; WBC, white blood cell count [NR 4.1-10.9 × 10^9^/L]; Plt, platelet count [NR 140-450 × 10^9^/L]; Lym, lymphocyte count [NR 20-50% of WBCs]; SLEDAI, systemic lupus erythematosus disease activity index.

### Generation of DCs from BM cultures and analysis of control BMDCs

Previous reports showed that the administration of FL to mouse BMCs generates large numbers of BMDCs *in vivo *and *in vitro *[20-22]. To determine whether FL had the same effects in humans, in addition to using GM-CSF/IL-4, we used FL to induce BMDC generation from both healthy donors and patients with SLE. Morphologic and phenotypic analysis of control BM mDCs and FLDCs are described subsequently.

#### Morphologic analysis of control BMDCs

**Figure 1:**
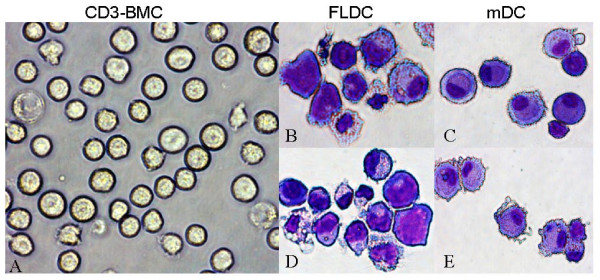
**SLE BMDCs cultured with FL alone (FLDCs) or GM - CSF + IL-4 (mDCs)**. Representative photographs of freshly isolated CD3^- ^BMCs **(a) **and May-Grunwald-Giemsa-stained cytospin preparations of immature FLDCs **(b)**, immature mDCs **(c)**, mature FLDCs **(d)**, and mature mDCs **(e)**. BMDCs, bone marrow-derived dendritic cells; CD3^- ^BMC, CD3^- ^bone marrow cells; FLDC, dendritic cells induced by FL; FL, FMS-like tyrosine kinase 3 ligand; GM-CSF, granulocyte macrophage-colony-stimulating factor; IL-4, interleukin 4; mDCs, myeloid dendritic cells.

As can be seen in Figure 1, cells cultured with either FL or GM-CSF/IL-4 became larger and developed typical dendritic cytoplasmic extensions. Figure 1a shows the morphology of CD3^- ^BMCs. Figure 1b and 1d depicts representative photomicrographs of immature and mature BM FLDCs, respectively, and Figure 1c and 1e shows immature and mature BM mDCs induced by GM-CSF/IL-4. No obvious differences were noted between immature and mature BM FLDCs or immature and mature BM mDCs. However, when compared with mDCs, some of the FLDCs had bigger nuclei, less cytoplasm, and fewer dendritic extensions.

#### Phenotyic analysis of control BMDCs

CD3^- ^BMCs and immature and mature BMDCs were stained with appropriate antibodies and analyzed with flow cytometry. No detectable CD3^+ ^cells and less than 1% of CD34^+ ^and less than 3% of CD19^+ ^cell impurities were noted in the DC-enriched populations (data not shown).

**Figure 2:**
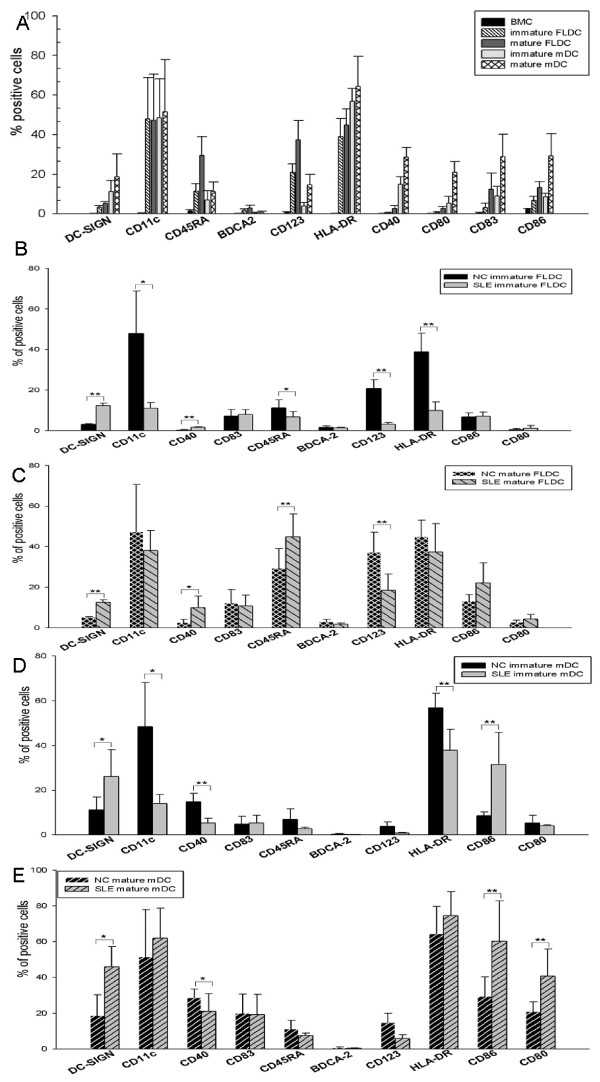
**Phenotypic analysis of control and SLE BMDCs induced with FL or GM-CSF + IL-4**. **(a) **Healthy control BMDCs induced with FL or GM-CSF + IL-4. When compared with BMCs, both immature FLDCs and mDCs expressed DC-SIGN, CD11c, CD45RA, HLA-DR, CD40, CD80, CD83, and CD86. Expression of these molecules was higher in mature FLDCs and mDCs. Comparing FLDCs and mDCs, both immature and mature FLDCs expressed BDCA-2 and CD123, whereas immature and mature mDCs expressed no or low levels of these molecules. **(b, c) **Immature FLDCs and mature FLDCs from normal controls and patients with SLE. SLE immature and mature FLDCs expressed higher levels of DC-SIGN, CD123, and CD40 but lower levels of CD11c and HLA-DR than did controls. **(d, e**) Immature and mature mDCs from normal controls and patients with SLE. SLE immature and mature mDCs expressed higher levels of DC-SIGN and CD86 and lower CD40 than did those of normal controls. *P < 0.05; **P < 0.01. Results are represented as mean ± SD of independent experiments of seven SLE patients and eight normal controls. BMDCs, bone marrow-derived dendritic cells; BMCs, bone marrow cells; FLDCs, dendritic cells induced by FL; mDCs, myeloid dendritic cells; FL, FMS-like tyrosine kinase 3 ligand; GM-CSF, granulocyte macrophage-colony stimulating factor; IL-4, interleukin 4.

Immature and mature BM FLDCs expressed increased levels of DC-SIGN, CD11c, HLA-DR, CD40, CD45RA, CD80, CD83, and CD86 when compared with CD3^- ^BMC (*P *< 0.05 for all surface markers). The BM FLDC-enriched population expressed higher BDCA-2 and CD123 counts when compared with CD3^- ^BMCs (*P *< 0.05 for BDCA-2 and *P *< 0.01 for CD123) (Figure 2a). With GM-CSF/IL-4, immature and mature BM mDCs showed significantly increased expression of DC-SIGN, CD11c, HLA-DR, CD40, CD45RA, CD80, CD83, and CD86 when compared with CD3^- ^BMCs (*P *< 0.05 for all surface markers). However, both immature and mature BM mDCs expressed lower levels of BDCA-2 and CD123 (Figure 2a).

**Figure 3:**
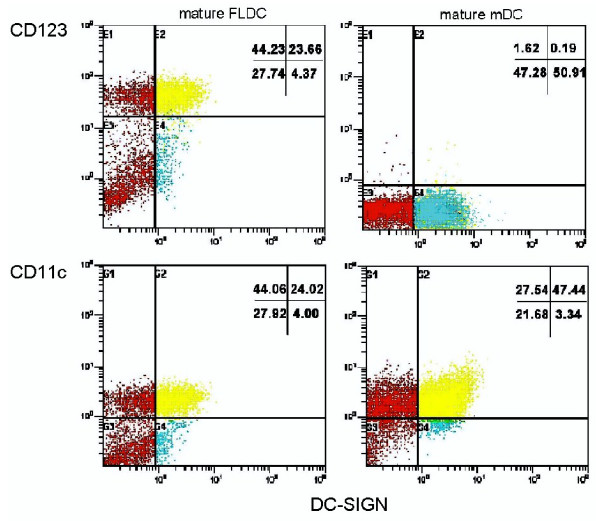
**Phenotypic analysis of mature FLDCs and mature mDCs**. Mature FLDCs expressed medium levels of CD11c and CD123, whereas mature mDCs expressed high levels of CD11c but only low levels of CD123. FLDCs, dendritic cells induced by FL; mDC, myeloid dendritic cells induced by GM-CSF + IL-4; FL, FMS-like tyrosine kinase 3 ligand; GM-CSF, granulocyte macrophage-colony-stimulating factor; IL-4, interleukin 4.

DC-SIGN^+ ^mature BM FLDCs included CD11c^+ ^(percentage of positive cells = 47.276 ± 23.354) and CD123^+ ^(percentage of positive cells = 37.236 ± 9.921) cell populations. However, mature DC-SIGN^+ ^BM mDCs expressed CD11c (percentage of positive cells = 51.45 ± 26.435; no significant difference was noted when compared with mature FLDCs), but lower CD123 (percentage of positive cells = 14.696 ± 5.177; *P *< 0.05 when compared with mature BM FLDCs) (Figure 3). Both immature and mature BM FLDCs expressed similar levels of CD11c and CD123, whereas immature BM mDCs expressed similar levels of CD11c but lower levels of CD123 expression when compared with mature BM mDCs (data not shown).

### Analysis of SLE BMDCs: comparison with control BMDCs

#### Phenotypic expression

**Figure 4:**
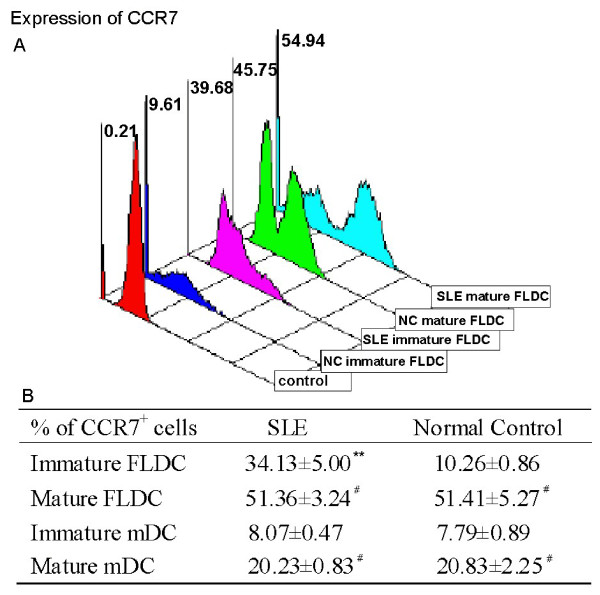
**SLE (n = 3) immature FLDCs expressed higher CCR7 than normal (n = 3) immature FLDCs**. **(a) **Mature FLDCs from both SLE patients and normal controls expressed higher-level of CCR7 than immature FLDCs. Immature FLDCs from SLE patients had a higher expression of CCR7 than did control. **(b) **Both control and SLE mature FLDCs and mDCs expressed higher levels of CCR7 than did immature FLDCs and mDCs, respectively. **P < 0.01; #*P *< 0.05. Results are expressed as mean ± standard deviation. NC, normal control; SLE, systemic lupus erythematosus; FLDCs, dendritic cells induced by FL; mDCs, myeloid dendritic cells induced by GM-CSF + IL-4; FL, FMS-like tyrosine kinase 3 ligand; GM-CSF, granulocyte macrophage-colony-stimulating factor; IL-4, interleukin 4.

Mature BM FLDCs and mDCs from both SLE patients and normal controls expressed increased CCR7 when compared with immature BM FLDCs and BM immature mDCs. However, SLE immature BM FLDCs expressed higher CCR7 than did controls. Figure 4 shows the CCR7 results from three patients with SLE and three normal controls.

SLE immature BM FLDCs expressed higher levels of DC-SIGN (SLE versus controls = 12.311 ± 1.286 versus 1.241 ± 0.262; *p *< 0.01) and CD40 (SLE versus controls = 1.629 ± 0.35 versus 0.312 ± 0.255; *P *< 0.01) than did normal controls. SLE immature BM FLDCs expressed lower levels of CD123 (SLE versus controls = 3.182 ± 0.956 versus 20.841 ± 14.258; *P *< 0.01), CD11c (SLE versus controls = 11.149 ± 2.777 versus 47.918 ± 20.843; *P *< 0.05), CD45-RA (SLE versus controls = 6.824 ± 2.663 versus 11.355 ± 3.925; *P *< 0.05) and HLA-DR (SLE versus controls = 9.908 ± 4.211 versus 38.906 ± 9.129; *P *< 0.01) than normal controls (Figure 2b).

SLE mature BM FLDCs expressed higher levels of DC-SIGN (SLE versus controls = 12.711 ± 1.104 versus 1.595 ± 0.424; *P *< 0.01), CD40 (SLE versus controls = 9.969 ± 5.729 versus 2.601 ± 1.582; *P *< 0.05) and CD45RA (SLE versus controls = 44.950 ± 11.225 versus 29.352 ± 9.699; *P *< 0.01) than normal controls. SLE mature BM FLDCs also expressed higher levels of CD86 than normal controls, although the difference was not statistically significant. SLE mature BM FLDCs expressed lower levels of CD123 (SLE versus controls = 18.542 ± 7.997 versus 37.236 ± 9.921; *P *< 0.01) than controls. The levels of CD11c and HLA-DR on SLE mature BM FLDCs were also lower than those in normal controls, but the difference did not reach statistical significance (Figure 2c).

SLE immature BM mDCs expressed higher levels of DC-SIGN (SLE versus controls = 26.110 ± 12.064 versus 11.179 ± 5.122; *P *< 0.05) and CD86 (SLE versus controls = 31.575 ± 14.177 versus 8.652 ± 1.667; *P *< 0.01) but lower levels of CD11c (SLE versus controls = 14.027 ± 4.169 versus 48.440 ± 19.606; *P *< 0.05), CD40 (SLE versus controls = 5.332 ± 2.052 versus 14.851 ± 3.756; *P *< 0.01) and HLA-DR (SLE versus controls = 37.833 ± 9.283 versus 56.862 ± 6.418; *P *< 0.01) (Figure 2d).

SLE mature BM mDCs expressed higher levels of DC-SIGN (SLE versus controls = 45.877 ± 11.245 versus 18.710 ± 11.521; *P *< 0.05), CD86 (SLE versus controls = 60.243 ± 22.651 versus 29.305 ± 10.987; *P *< 0.01) and CD80 (SLE versus controls = 40.601 ± 15.245 versus 20.970 ± 5.445; *P *< 0.01) but lower levels of CD40 (SLE versus controls = 20.972 ± 9.855 versus 28.599 ± 4.847; *P *< 0.05) than controls (Figure 2e).

#### Production of IFN-α

In SLE, both immature and mature BM FLDCs produced detectable levels of IFN-α, whereas immature and mature BM mDCs did not. Furthermore, mature BM FLDCs produced higher levels of IFN-α when compared with immature BM FLDCs (mature versus immature BM FLDCs = 65.59 ± 25.45 versus 10.52 ± 5.60 pg/ml; *P *= 0.022). Because IFN-α is produced primarily by pDCs, these results further suggest that BM FLDCs comprise a subpopulation of pDCs that are capable of responding to CpG ODN stimulation.

In normal controls, no IFN-α was detected in the culture supernatants of either immature or mature BM FLDCs and mDCs.

#### Mixed lymphocyte reaction

**Figure 5:**
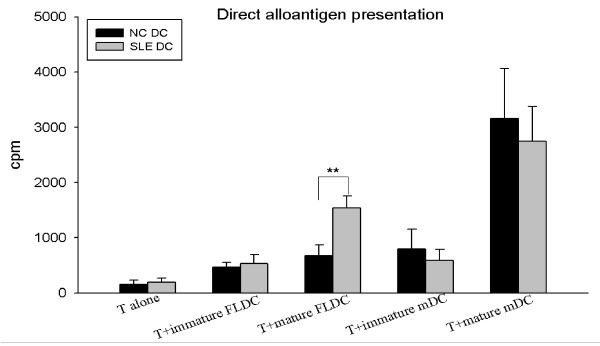
**Allogeneic T-cell proliferation induced directly by DCs**. Bar graphs representing allogeneic T-cell proliferation induced by BMDCs from five patients with SLE and 11 normal controls. Both SLE immature and mature FLDCs induced higher T-cell proliferation, whereas SLE mDCs induced lower T-cell proliferation when compared with normal controls. **P < 0.01. Results are represented as mean ± SD of independent experiments. NC, normal control; SLE, systemic lupus erythematosus; FLDCs, dendritic cells induced by FL; mDCs, myeloid dendritic cells induced by GM-CSF + IL-4; FL, FMS-like tyrosine kinase 3 ligand; GM-CSF, granulocyte macrophage-colony-stimulating factor; IL-4, interleukin 4.

Both immature and mature SLE FLDCs expressed a higher ability to induce T-cell proliferation when compared with normal controls. As with normal control mature mDCs, SLE mature mDCs induced higher T-cell proliferation than did immature mDCs. SLE mature mDCs tended to induce lower levels of T-cell proliferation when compared with control mature mDCs. However, the difference was not statistically significant (Figure 5).

## Discussion

The immunopathogenesis of SLE is complex and is characterized by multiple T- and B-cell abnormalities. Central to these changes are believed to be altered functions of DCs, the most important APCs [3,4,14,23-25].

Peripheral tolerance is believed to be broken in SLE [26]. DCs, which have a significant role in maintaining peripheral tolerance, have been found to be defective and proposed to be important in the development of autoimmunity in SLE [3]. Of the two DC subsets, pDCs are thought to have a central role in SLE pathogenesis through the production of IFN-α, which has a pivotal role in inducing SLE [27,28]. Although controversial, the number of pDCs in peripheral blood is aberrant when compared with that in normal controls [4,24,29].

mDCs also have been found to be abnormal in SLE [24,30,31]. Patients with this condition have deficient number of mDCs [4] and monocyte-derived DCs that exhibit abnormal phenotypes and functions [32]. Decker *et al*. [14] reported that monocyte-derived DCs from SLE patients expressed high levels of CD86 and produced increased quantities of IL-6 on stimulation.

Previous studies focused mostly on PBMC-derived DCs or DCs that are directly isolated from peripheral blood [24,33]. It is not known whether defects of these DCs are secondary to (a) DC precursor deficiency; (b) microenvironmental changes in the bone marrow during DC development; or (c) microenvironmental changes or after Ag capture in the peripheral blood and the site of tissue injury. Although murine BMDCs have been studied previously, data on the characteristics and function of human BMDCs in patients with SLE is scarce. It is for this reason that we compared the phenotypic and functional characteristics of BMDCs from SLE patients and healthy individuals.

Traditionally, DCs are generated *in vitro *by using GM-CSF/IL-4 [34-37]. However, this method induces only mDC generation. In mouse studies, FL has been reported to be capable of inducing pDC development [21,22,38,39]. Therefore, in our experiments, besides using the traditional GM-CSF/IL-4 culture method to study BM-derived mDCs, we also applied FL to induce BM cells to develop into DCs (which we defined as BM FLDCs), which showed features of both mDCs and pDCs and allowed us to study BM-derived pDCs indirectly.

In the present study, we confirmed that human CD3^- ^BMCs could be induced to become DCs, with FL as the only growth factor. Consistent with previous studies, BM FLDCs had an increased expression of DC-SIGN, a DC marker, and some costimulator molecules including CD40, CD80, and CD86 when compared with BMCs and the classic DC-culture system involving GM-CSF/IL-4 [35,37] which induced mainly mDC development. FL appeared to induce both mDC and pDC development. During differentiation, some of the BM FLDCs expressed phenotypic characteristics (BDCA-2, CD123) similar to those identified in pDCs, whereas others expressed CD11c, which is normally seen in mDCs. In addition, we found that FL-generated mDCs and pDCs existed in a ratio of 1:1. This is consistent with findings reported in previous studies on murine FLDCs derived from BM and peripheral blood [21,39,40].

To study the phenotypic and functional characteristics of BMDCs at different stages of differentiation, various agents were used to stimulate the maturation of these cells. For immature BM mDCs, TNF-α/LPS were used to stimulate their maturation. However, TNF-α/LPS have not been used to stimulate immature pDCs previously. In this study, therefore, we used CPG ODN2006/CPG ODN2216 plus TNF-α/LPS to stimulate BM FLDCs. After stimulation, BM FLDCs showed increased expression of CD80, CD86, CD40, and CD83, indicating that these cells could be stimulated to maturity efficiently by this method.

Results from our study showed that SLE BMDCs have defective phenotypic expression and function when compared with those from healthy subjects. CCR7 is a chemokine receptor that is preferentially expressed by mature DCs and is important for DC migration [41,42]. In our study, we found that immature BM FLDCs from SLE patients expressed higher levels of CCR7 than did those from normal controls, indicating that these cells may have a stronger ability to migrate. Because no obvious differences in CCR7 expression were found between SLE and normal immature BM mDCs, the higher expression of this chemokine receptor on SLE immature BM FLDCs should have been contributed by the pDC population among these cells. The higher CCR7 expression may allow SLE pDCs to migrate into lymph nodes where they could interact with T lymphocytes. This may also partly explain the low number of pDCs found in the peripheral blood of SLE patients in some previous studies [43,44]. However, to confirm that SLE pDCs have a higher ability to migrate, further studies using an *in vitro *migration assay are needed.

During DC maturation, HLA-DR expression is upregulated. However, in patients with SLE, both BM FLDCs and mDCs expressed lower levels of HLA-DR when compared with controls. Previous studies have suggested that deficiency in HLA-DR expression might be the cause of increased susceptibility of patients with SLE to various infections [32]. In our study, we found that SLE immature and mature BM mDCs failed to stimulate T-cell proliferation as efficiently as did those obtained from normal controls. This may be explained by their lower expression of HLA-DR. However, this was not true for BM FLDCs. Both immature and mature BM FLDCs stimulated higher T-cell proliferation compared with controls. As BM FLDCs include a mixed population of pDCs and mDCs and because mDCs did not stimulate T-cell proliferation efficiently, the effects of BM FLDCs on T-cell proliferation may be attributed to the pDC subpopulation of BM FLDCs. This effect may be related to the higher expression of CD40, CD80, and CD86 on SLE BM FLDCs than in controls.

To evaluate whether BM FLDCs comprise a subpopulation of pDCs and whether SLE BM FLDCs had higher pDC activity, we measured the level of IFN-α by using ELISA in the supernatants of BM FLDC and mDC cultures. IFN-α is produced mainly by pDCs, and its serum level has been reported to be higher in patients with SLE [27,45]. In this preliminary analysis, we found that SLE BM FLDCs produced detectable IFN-α, whereas normal BM FLDCs did not. Furthermore, mature SLE BM FLDCs produced higher levels of IFN-α than did immature SLE BM FLDCs. Neither SLE nor control BM mDCs produced detectable IFN-α. These findings further confirmed that BM FLDCs consisted of both mDCs and pDCs, as per earlier suggestion. It also confirmed that pDCs were the more active of the two types of DCs in SLE and may be the major culprit in inducing autoimmunity in this condition. It should be noted that IFN-α measurement was performed only in the BMDC culture supernatants from a few subjects; further studies are needed to confirm this finding. It is interesting to note that a recent study showed that peripheral-blood pDCs from patients with chronic SLE had decreased *in vitro *IFN-α-producing capacity and were desensitized to TLR9 stimulation [13]. These data, plus those reported previously [3,7,8,13,14] and our current data on BMDCs provide further important insight into the role(s) of pDCs in SLE pathogenesis. We hypothesize that pDCs are the dominant DCs during their development in the BM. These IFN-α-producing cells induce the development of SLE. However, they may subsequently become deficient, with reduced IFN-α producing capacity and tolerance to TLR9 stimulation, probably as a result of chronic and persistent exposure to DNA-containing immune complexes in the peripheral environment, which are a hallmark of SLE.

Some limitations to our study exist. First, the number of subjects studied was small. Second, our findings may not be generalized to all patients with SLE, as the patients recruited in this study all had some form of cytopenia or fever requiring further investigations, including a BM examination. Patients with other lupus manifestations were not recruited, as we considered it unethical to perform a BM examination in these subjects. Third, most of the patients studied were receiving some form of treatment, including immunosuppressive agents. It is, therefore, not possible to confirm whether the BMDC changes were a result of the underlying disease or that of the various lupus medications. It should, however, be noted that the majority of these patients had active lupus disease-related cytopenia or fever despite drug treatment; it is therefore tempting to suggest that our findings reflect the true role of BMDCs in lupus disease pathogenesis. Future studies should aim to recruit treatment-naïve or newly diagnosed patients with SLE. However, this will have to involve the collaboration of multiple lupus research units. It has taken the authors more than 2 years to recruit 13 suitable patients from a cohort of more than 500 patients for the purpose of this study. Alternately, future studies may include culturing control BMDCs *in vitro *with the various immunosuppressive drugs to evaluate whether they acquire a similar phenotype to the one described in this study.

Our study also did not examine whether the BMDC changes were intrinsic defects or secondary to microenvironmental changes in the BM, including the cytokine milieu in our SLE patients. This should be evaluated in future studies. DC precursors in the bone marrow are mainly CD34^+ ^stem cells [35]. Sun and colleagues [46] recently reported that CD34^+ ^stem cells from patients with SLE had abnormal expression of CD166 and CD123 and that these abnormalities correlated with the overall lupus disease activity. Mesenchymal stem cells (MSCs), an important compartment in the BM, are believed to be able to affect DC generation, although previous findings have been controversial [47,48]. Deficient MSCs from patients with SLE have been reported [49], but whether MSC may affect BMDC generation and functions requires further detailed studies. In addition, the phenotypes and functions of DCs from patients with SLE could be altered by genetic defects in cell lineage, or as a result of factors capable of inducing their differentiation and maturation. Previous studies have shown higher levels of multiple cytokines in the BM, some of which may be pathogenic in SLE [50]. DCs from patients with SLE could bear genetic alterations that made them prone to maturation under abnormal conditions, or they may be normal cells with an abnormal phenotype and behavior induced by the bizarre microenvironment from which they were obtained. Further investigations are required.

## Conclusions

DCs have a significant role in antigen processing and presentation, leading to naïve T-cell stimulation or the development of immune tolerance. Defects in DCs may lead to an imbalance of the immune system, including alterations of T and B cells, and may lead to autoimmunity, such as the development of SLE. Here we suggest that both BM mDCs and FLDCs from patients with SLE are defective. Our results are in accordance with previous studies that suggested that mDCs are deficient in patients with SLE and may contribute to their susceptibility to infections, but pDCs, which are part of FLDCs, are the major culprit in SLE.

## Abbreviations

APC: antigen-presenting cells; BM: bone marrow; CpG ODN: oligodeoxynucleotide [ODN] containing unmethylated CpG motifs; DCs: dendritic cells; FL: FMS-like tyrosine kinase 3 (Flt3)-ligand; FLDC: dendritic cells cultured with FL; GM-CSF: granulocyte-macrophage colony-stimulating factor; IFN-α: interferon alpha; IL-4: interleukin-4; LPS: lipopolysaccharide; mDC: myeloid dendritic cells; MHC: major histocompatibility complex; PBMCs: peripheral blood mononuclear cells; pDC: plasmacytoid dendritic cells; R: responder cell; S: stimulator cell; SLE: systemic lupus erythematosus; SLEDAI: systemic lupus erythematosus disease activity index; TNF-α: tumor necrosis factor-α.

## Competing interests

The authors declare that they have no competing interests.

## Authors' contributions

CSL was responsible for the strategy, planning, funding, and the integrity of the study. He also supervised data collection, statistical analysis, and manuscript drafting. YJN was responsible for the strategy of the study and conduct of all experiments. She collected and analyzed the data and drafted the manuscript. MYM and AKWL provided the bone marrow samples and supervised data collection. GCFC contributed to manuscript preparation. OJ, SK, and AC contributed to data collection and analysis and to technical support. All authors were actively involved in the drafting and the final approval of the manuscript.
